# A Martini 3 coarse-grain model for the simulation of the photopolymerizable organic phase in dental composites

**DOI:** 10.1039/d2ra00732k

**Published:** 2022-04-20

**Authors:** Alexander Hochwallner, Jürgen Stampfl

**Affiliations:** Institute of Materials Science and Technology, TU Wien Getreidemarkt 9 1060 Vienna Austria alexander.hochwallner@tuwien.ac.at

## Abstract

Light-hardening dental composites can be used in a large number of applications in restorative dentistry. They are based on photopolymerizable resins, which are highly relevant also in other industries like 3D printing. Much effort is therefore being put into developing and optimizing photopolymers. Currently used photopolymers still have limitations regarding mechanical properties, shrinkage and leaching of uncured monomers. These issues are strongly linked to the network structure of the polymer and are usually addressed using trial and error methods. Therefore, it is of interest to have a model for the network structure of such materials and to have a tool to facilitate scientific progress and the development of high-performance photopolymers. This work presents a coarse grain model of Bis-GMA/TEGDMA formulations and their corresponding networks, following the Martini 3 guidelines and using a simulated polymerization algorithm. The model proved to reproduce the densities and volumetric shrinkage values found in the literature well. Furthermore, it was possible to estimate the final double bond conversion of the polymer material. Martini's building block-like design makes it easy to extend the model to other monomers in the future.

## Abstract 2

A coarse grain model of Bis-GMA and TEGDMA is presented and used to study aspects relevant to dental composites. It was found that the simulated polymerization shrinkage and double bond conversions are in good agreement with experimental data.

## Graphical abstract

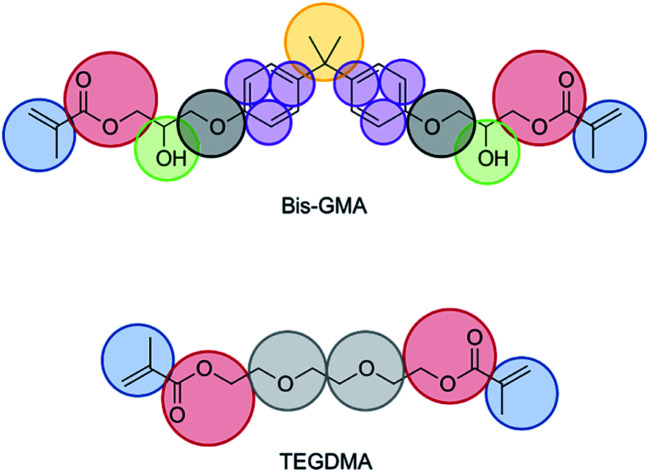

## Introduction

**Scheme 1:**
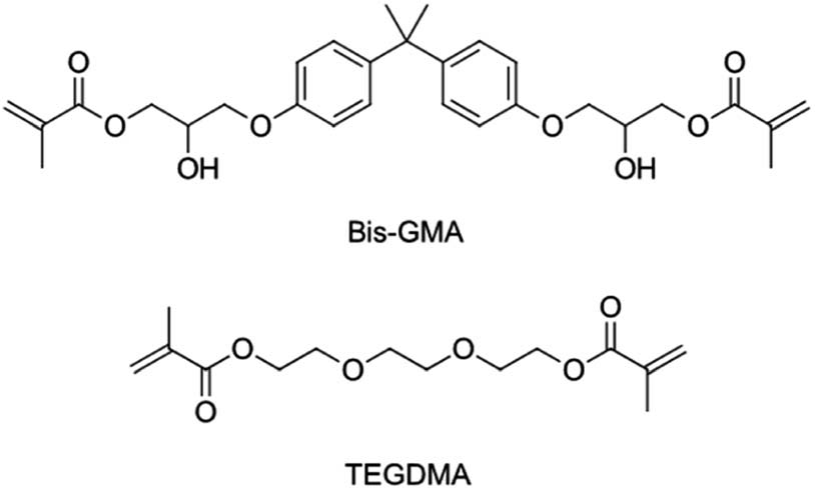


Light-hardening composites as restorative materials in dentistry are very popular. They are used to replace amalgam as the default choice for dentists, and patients.^1^ Dental composites consist of an organic matrix, which solidifies upon photochemical initiation, and inorganic fillers.^2^ The organic phase most commonly contains Bis-GMA diluted by TEGDMA.^3^ Both are bifunctional methacrylate substances. The molecular structures are shown in Scheme 1.

For a composite to be used in restorative dentistry, it must fulfil several challenging requirements. The mechanical properties but also chemical stability are of high importance. Additionally, polymerization shrinkage poses a limitation to the applicability of light-cured polymer networks.^4,5^

In order to establish safe and reliable curing schemes for the application of dental compounds, the properties of the organic phase need to be investigated thoroughly regarding the composition of resins and the properties in relation to the polymerization reaction.

Photopolymer resins are not only being used in dental applications. They are also used extensively in additive manufacturing, which has the potential to become a key technology in the future, provided the material properties meet the requirements.^6^ Much effort is therefore being put into synthesizing new 3D printing materials for a variety of applications. Polymerization shrinkage is also an issue in additive manufacturing as it can lead to warpage and residual stresses.^7^ Both of which compromise the usability of 3D printed parts.

Developing and optimizing photopolymer resins is a time-consuming task, which includes mixing of the components, photochemical curing, testing and characterization. In order to optimize a resin composition, the outlined sequence of steps has to be repeated with different ratios of the ingredients and different curing schemes. This leads to a high number of samples to be analyzed. Hence, it is of interest to establish a method that can screen a large part of the compound space automatically or at least semi-automatically.

Another limitation to the purely experimental approach is that no method is available to get direct information on the network structure. It is evident that the constitution of the cured polymer network is integral to the properties of the final material. Therefore, establishing structure–property relations is of high relevance. Additional information on the network structure may make it easier to relate material properties to the molecular network structure obtained with different curing conditions, giving way to optimizing dental composites and 3D printing resins. This work aims to explore the possible usage of molecular simulations for the investigation of photopolymer resins. The technologically highly relevant mixture of Bis-GMA with TEGDMA was chosen as a test system. The transferability of the modeling approach to molecules relevant in additive manufacturing is a key motivation for this work.

Molecular dynamics simulation is a method to investigate a material on the molecular level with possibilities to automate the tasks.^8^ To get accurate results from molecular dynamics simulations, the force field must be adequate, and system sizes and time scales must be large enough.^9^ To satisfy these conditions, coarse grain force fields for molecular systems are under constant development. A coarse grain model maps groups of individual atoms to one coarse grain bead. The underlying chemical group then defines the inter-particle interactions as effective interactions.^10^ The reduction of the degrees of freedom by grouping atoms to beads leads to computationally less expensive simulations. This enables the simulations of larger systems over a longer period of time compared to all-atom simulations.

One widely used coarse grain potential that provides recommended mapping schemes and interparticle potentials is Martini.^11^ The strength of the Martini force field is, on the one hand, its easy applicability, and on the other hand, its verified accuracy in numerous chemical problems.^12^ The Martini beads are designed to be used as building blocks and can be applied to a variety of different molecules.^12,13^ This building block approach makes it perfectly suitable to efficiently explore the space of photopolymer materials.

Compared to previous Martini models, Martini 3 offers more bead types, and the mapping scheme was changed from centre of mass mapping to centre of geometry mapping. This change leads to better reproduction of packing densities.^14^

This work presents a molecular dynamics model following the Martini 3 mapping scheme to simulate the polymer network of the organic phase in dental composites. Next to proper representations of the monomers, this requires a close to reality network structure. To achieve this, a simulated polymerization algorithm called Polymatic^15^ is applied. It is used to establish chemical bonds between beads that represent the reactive groups of the monomers. This is done by employing a cut-off criterion, which means that a bond is formed if a bead labelled as active and a bead labelled as reactive are within a certain distance to each other. The active label is then passed on to the reaction partner to allow for chain-growth polymerization.

The model is verified by comparing monomer densities and curing shrinkage to values reported in literature. A comparison to experimental double bond conversions is also presented to give an outlook on the predictive value of a reactive model to study photopolymer resins. The present model should serve as a basis for future extensions with arbitrary monomers of interest.

## Computational details

For all-atom simulations, GROMACS^16^ was used. The run parameters were obtained from the Martini web page^17^ and are in accordance with ref. 13. Only the constraints setting was changed from all-bonds to h-bonds. All-atom OPLS force field files were generated using LigParGen^18–20^ following ref. 17. The simulation box was prepared to contain a single molecule of interest, diluted in 500 molecules of TEGDMA. Energy minimization, equilibration, and production run were done as described in ref. 17. The mapping files necessary to map the trajectories from the all-atom representation to the coarse-grain representation were generated using CGbuilder.^17,21^ The coarse grain runs needed to obtain the coarse grain distributions were also done with GROMACS^16^ and run files taken from ref. 17. The time step was set to 20 fs. The remaining parameters were used as provided. All distributions were evaluated using the files provided in ref. 17.

The simulated polymerization was done using Polymatic^15^ which sequentially checks the defined bonding criterion, updates the topology if a bond was formed, and calls LAMMPS^22^ for energy minimization and molecular dynamics run. The simulation box for the polymerization simulation was prepared with the packing algorithm that is delivered with Polymatic.^15^ First, the active monomers that acted as initiators were randomly added to the box. Then Bis-GMA molecules were added. The remaining TEGDMA molecules were added last. Then energy minimization was done, and the system was equilibrated at 298 K and hydrostatic pressure of 1 bar for 80 000 timesteps with 20 fs. Polymatic^15^ was set to call LAMMPS^22^ for energy minimization, and molecular dynamics run for 40 000 timesteps after ten bonds were successfully formed. A 20 000 timesteps run is performed if no bond was formed, and the bond formation is attempted again. The cut-off for bond formation was set to 5.5 Å. The simulation was terminated if no bond was formed after 40 attempts. During all runs, a temperature of 298 K and a pressure of 1 bar were maintained. The Shake algorithm was used because LAMMPS does not have the Lincs algorithm implemented. For that, one angle and two bonds are constrained in every aromatic ring in the system. The number of molecules in each formulation was (0;559), (200;447), (300;391), (400;335), (500;279), (600;223), (700;168), (800;112), (1000;0). The number before the semicolon represents TEGDMA. Temperature sweeps were performed over one million timesteps from 150 K to 500 K.

## Coarse graining

### Bead assignment

**Fig. 1:**
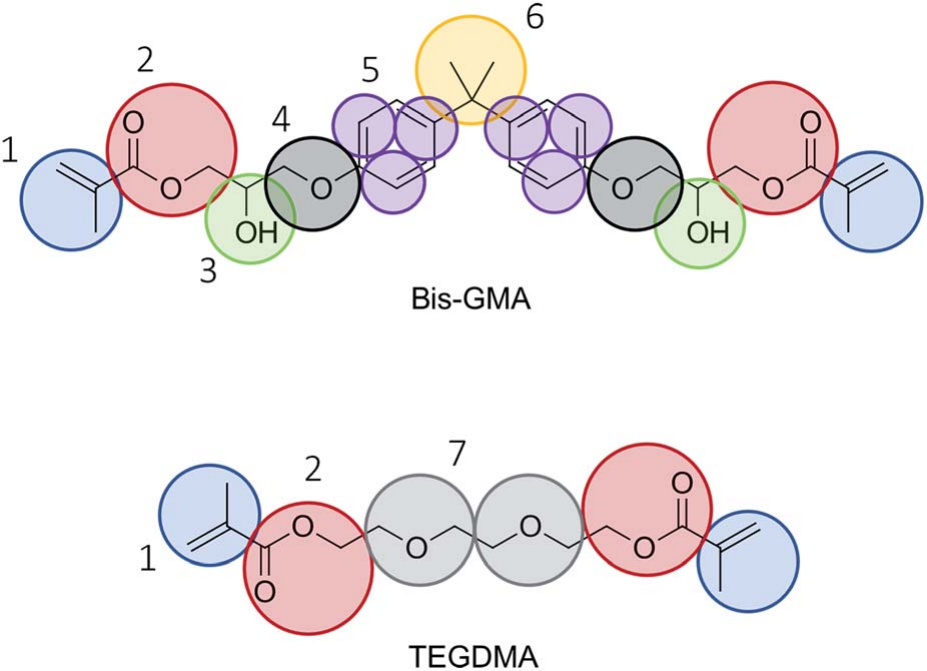
Mapping scheme. Choices for Martini 3 bead types are given in Table 1.

The mapping of the atoms into coarse grain beads was done as indicated in Fig. 1. Care was taken to have two, three, or four heavy atoms grouped into tiny, small, and regular beads, respectively. The bead type of bead number 4 was chosen to be TN2a in accordance with methoxybenzene as proposed in ref. 23. The remaining bead types were selected to be in accordance with the default choice of bead types as presented in ref. 14 and 24. The bead choices are summarized in Table 1.

**Table 1:** Choices of standard Martini 3 bead types for mapping Bis-GMA and TEGDMA

Bead number	Martini 3 bead type
1	SC4
2	N4a
3	TP1
4	TN2a
5	TC5
6	SC2
7	SN3a

### Bonded interactions

The coefficients for the bonded interactions were set to best match the distributions of the all-atom simulation. The values for the harmonic bond coefficients are given in Table 2. The harmonic bond potential has the following form:
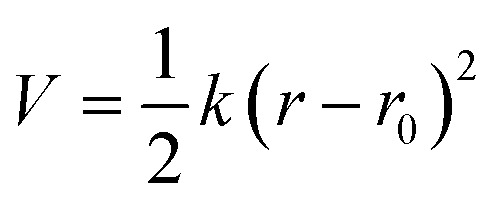
where *k* is the force constant, *r* is the distance between bonded beads and *r*_0_ is the equilibrium distance. Note that the bonds between the beads forming the aromatic structure are modelled using constraints as the bond length distribution is narrow.^23^ Two different bond lengths were used between type 5 beads. A longer bond length was used between type 5 beads bonded to bead type 4. Table 3 shows the coefficients for the harmonic angles. To reproduce the shape of the all-atom simulation, a relatively stiff angle was necessary in Bis-GMA for the angle with a type 6 bead as the central bead. The harmonic angle potential looks as follows:
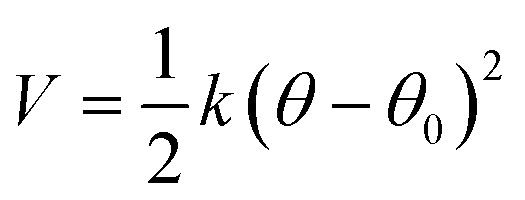
where *θ* is the three-body angle between bonded beads and *θ*_0_ is the equilibrium three-body angle.

**Table 2:** Harmonic bond coefficients

Bead–bead	Distance (nm)	Force constant (kJ mol^−1^ nm^−2^)
1–2	0.355	35 000
2–3	0.23	5000
3–4	0.24	28 000
4–5	0.3	15 000
5–5*^constrained^	0.229	
5–5*^constrained^	0.197	
5–6	0.271	35 000
2–7	0.345	6000
7–7	0.37	9000

**Table 3:** Harmonic angle coefficients

Bead–bead–bead	Angle (deg)	Force constant (kJ mol^−1^ rad^−2^)
1–2–3	108	110
2–3–4	76	120
3–4–5	130	40
4–5–5	120	80
5–5–6	140	130
5–6–5	70	700
1–2–7	115	100
2–7–7	124	100

**Fig. 2:**
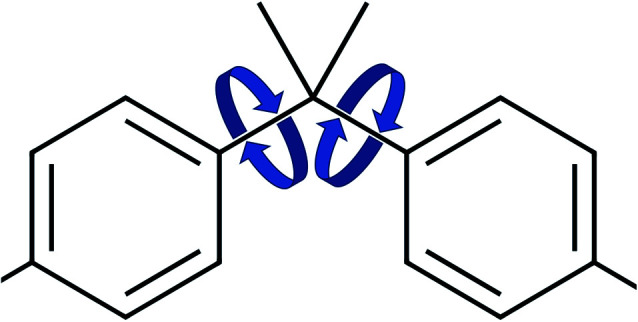
Dihedral rotations of the aromatic rings.

**Fig. 3:**
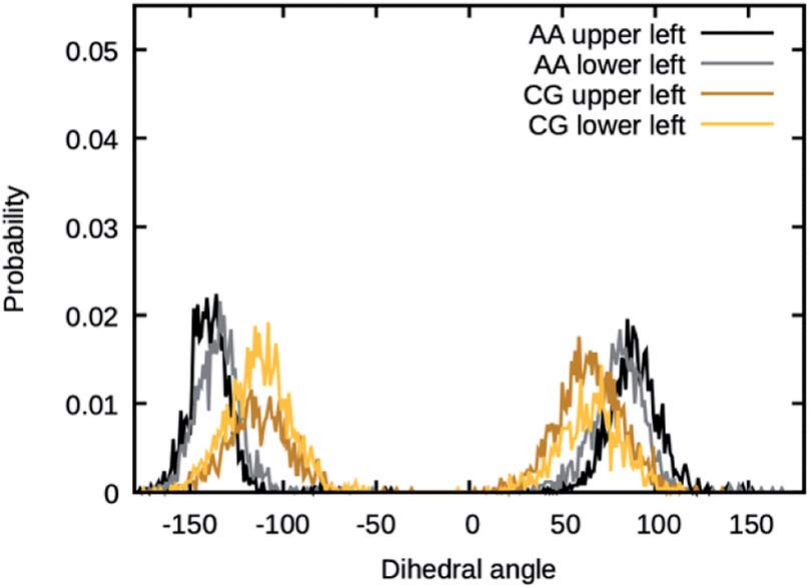
Dihedral angles in Bis-GMA, which show two peaks in the distribution.

**Fig. 4:**
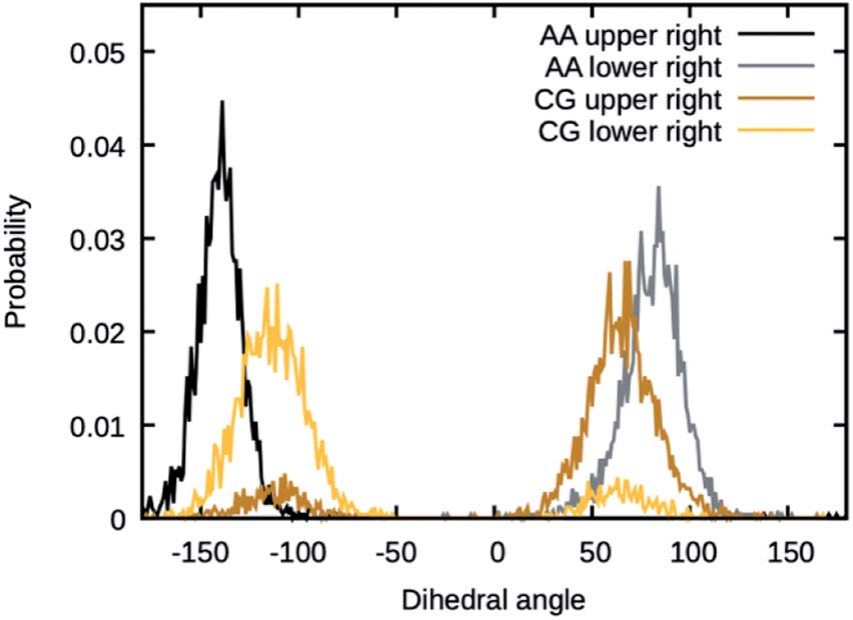
Dihedral angles in Bis-GMA, which show one peak in the distribution.

The values for the dihedral coefficients used are presented in Table 4. Special care was taken to reproduce the angular arrangement of the aromatic rings with respect to each other (see Fig. 2). The all-atom simulation showed that the distribution of the dihedrals that describe the aromatic ring's rotation are bimodal and not independent of each other. One of the rings in a Bis-GMA molecule resides in one of two possible angular arrangements, while the other one is present in both arrangements with equal probability. This behavior could be reproduced in the coarse grain representation using two dihedral angles per aromatic ring and carefully adjusting the force constant. A too high force constant would entrap left and right aromatic dihedrals into one of the possible configurations. A force constant that is too low would result in equal probability of the configurations. Fig. 3 shows the dihedral distributions of the central dihedrals in a Bis-GMA molecule with equal probability of both configurations. Fig. 4 shows the dihedrals at the second aromatic ring. Here one can see that only one configuration is occupied. In the case of the all-atom simulation, the distributions are located around a single angular value. In comparison, the coarse grain simulation also shows some probability to be present in the other configuration. However, the maximum is clearly located around a single angular value which is a good approximation of the all-atom simulation.

**Table 4:** Dihedral coefficients

	Angle (deg)	Energy (kJ mol^−1^)	Multiplicity
1–2–3–4	0	35	1
5–5–7–8	313	8.46	2
1–2–7–7	0	2	3
2–7–7–2	0	2	3

In order to keep the aromatic rings planar, improper dihedrals were used. The values for the dihedral coefficients are given in Table 5. The following functional form was used for the dihedral potential and improper potential:*V* = *k*(1 + cos(*n*ϕ − *ϕ*_0_))where *k*, in this case, is the energy constant, *ϕ* is the dihedral or improper angle and *n* is the multiplicity.

**Table 5:** Improper dihedral coefficients

	Angle (deg)	Energy (kJ mol^−1^)	Multiplicity
4–5–5–5	0	65	1
5–5–5–6	180	100	1

## Simulated polymerization

In order to obtain a realistic network structure of the polymerized material, a simulated polymerization approach was chosen. To do this, Polymatic^15^ was used. This code uses a simple cut-off criterion to decide whether or not a bond will be established between two beads. All beads in a simulation representing a reactive group are labelled as such. The beads that currently bear the radical character are labelled as active. If two beads labelled as active and reactive are within the cut-off distance to each other, a bond is established between them. The active label is then passed on to the reaction partner for the chain polymerization to proceed. After a successful bond formation, the backbone potential was updated. To evaluate the additional interactions necessary for the polymer, a TEGDMA 4-mer was parametrized using the same method as described above. For the sake of numerical stability, no backbone dihedrals were set. The backbone angle was set to 350 kJ mol^−1^ rad^−2^ at an equilibrium angle of 125 deg. The backbone-sidechain angle was set to 50 kJ mol^−1^ rad^−2^ at an equilibrium angle of 80 deg, and the backbone bond was set to 35 000 kJ mol^−1^ nm^−2^ and bond length of 0.31 nm.

## Results and discussion

### Simulated density

**Fig. 5:**
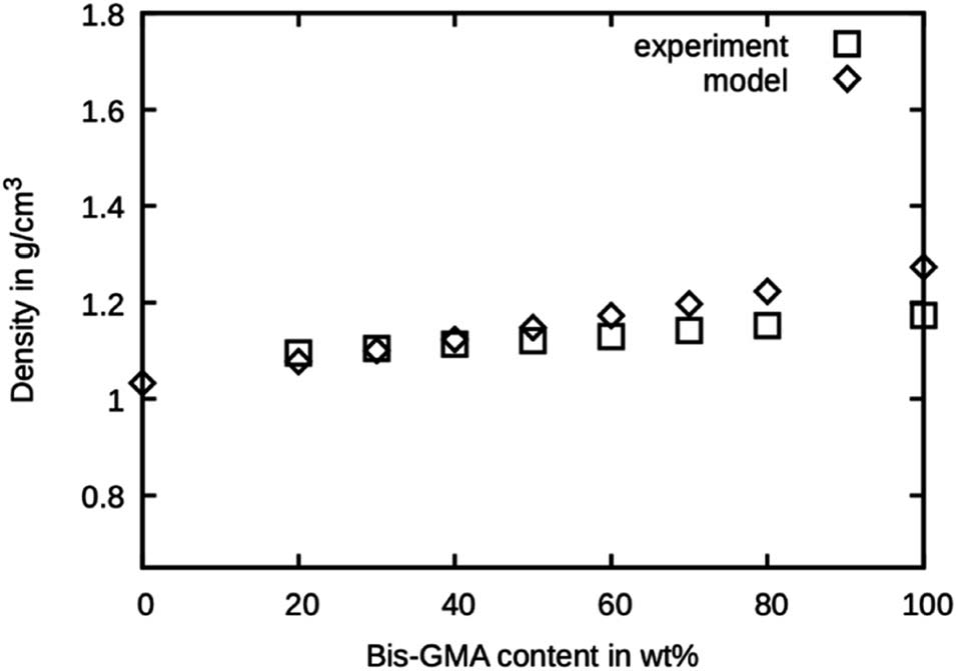
Densities as measured by Dewaele *et al.*^25^ compared to the presented model over the Bis-GMA content.

The simulated densities for the respective compounds are presented in Fig. 5. The values for the mixtures with little Bis-GMA content are in good agreement with the values reported by Dewaele *et al.*^25^. With higher Bis-GMA content, the deviation increases. A possible explanation for this is that Bis-GMA was parametrized with respect to a system of a single Bis-GMA molecule in a box of TEGDMA as a solvent. As a result, the Bis-GMA – Bis-GMA interactions are not accurately captured, whereas Bis-GMA – TEGDMA and TEGDMA – TEGDMA interactions are properly modelled. Another possible source of deviation is the choice of MARTINI 3 bead types. Definition of custom bead types backed by free energy calculations could potentially improve the overall model.^26^ For the sake of simple addition of other molecules later on, only standard beads were used in this work. Note that for calculating the model densities, the actual molecular weights of Bis-GMA and TEGDMA were used, not the standard MARTINI masses. For all simulation runs, standard MARTINI masses were used.

### Shrinkage of simulated material

**Fig. 6:**
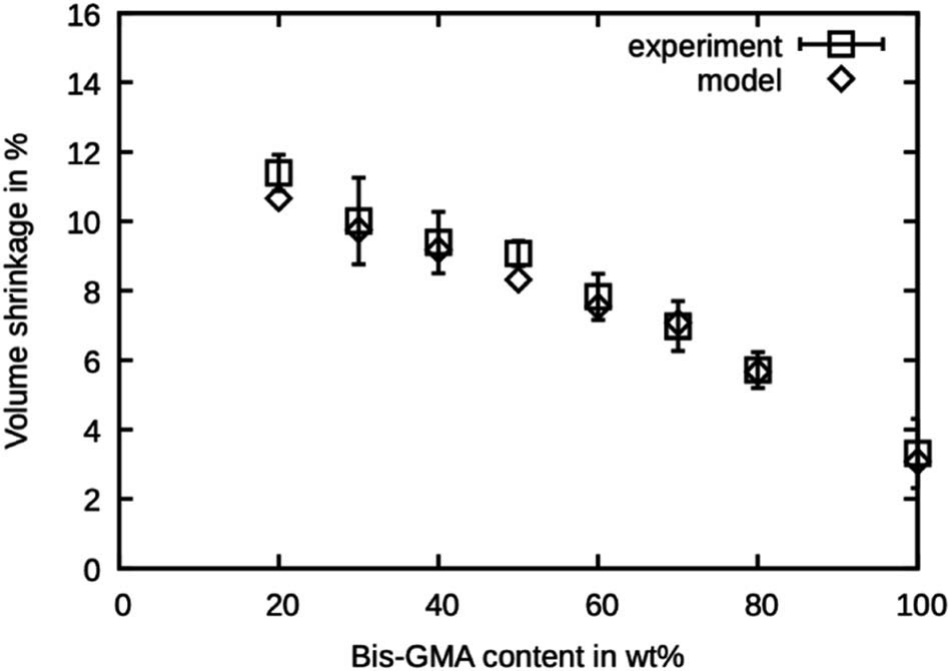
Polymerization shrinkage as measured by Dewaele *et al.*^25^ compared to the simulated values over the Bis-GMA content.

The shrinkage was determined as the volumetric decrease of the simulation box after the degree of conversion has reached the values reported by Dewaele *et al.*^25^ The values are presented in Fig. 6 and are in excellent agreement with the experimental values. The model is within the reported standard deviation except for the compound with 20 wt% Bis-GMA and 50 wt% Bis-GMA content. The deviation in densities of the high Bis-GMA compounds does not seem to influence the precision of the shrinkage values negatively.

### Double bond conversion

**Fig. 7:**
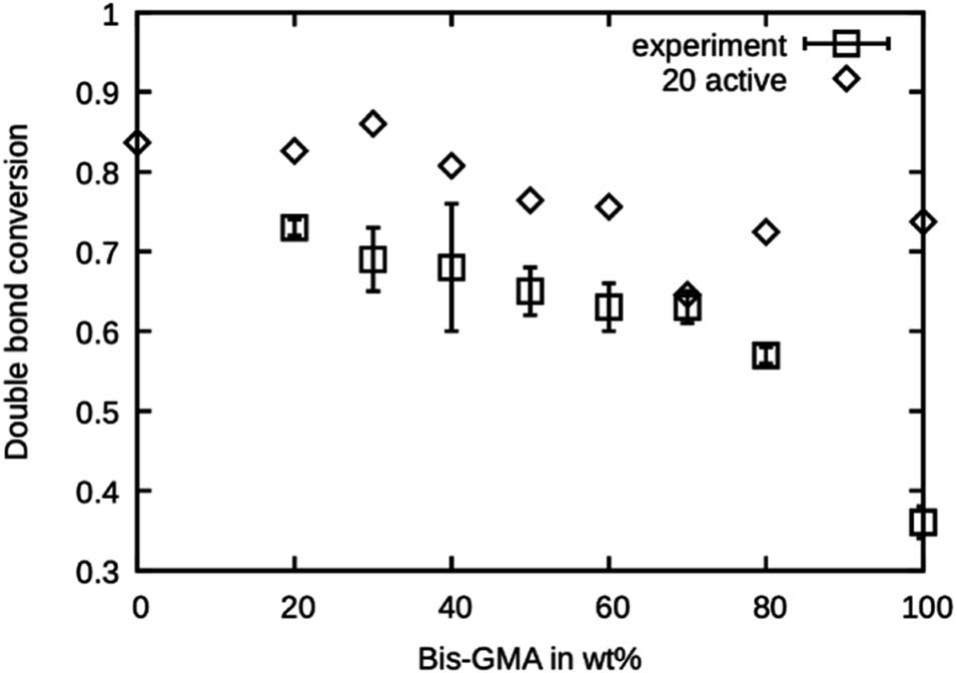
Double bond conversion as measured by Dewaele *et al.*^25^ compared to the double bond conversion of the model with 20 active sites after the termination criterion was reached over the Bis-GMA content.

The simulated polymerization method offers the possibility of learning about the investigated material's reaction kinetics prior to material synthesis. This can be a valuable information in resin development because the degree of polymerization greatly influences the material properties and possibly limits the applicability of a photopolymer. It was investigated if the presented simulated polymerization procedure allows for an estimation of the final double bond conversion of various Bis-GMA – TEGDMA compositions. The results of the simulated polymerization using 20 active sites are shown in Fig. 7, together with the experimental values reported by Dewaele *et al.*^25^ It can be seen that the simulation yields too high values for the case of 20 active propagation sites. Only at a Bis-GMA content of 70 wt% is the agreement reasonable. The agreement for pure Bis-GMA is not good. Generally, the simulated double bond conversion values are expected to inherit more uncertainties, coming from the fact that the condition for polymerization termination was chosen more or less arbitrarily. However, the trend of the values from the experiment is reflected in the simulation, showing that the model can reasonably accurately simulate the mobility of the reaction partners during curing of the resin.

**Fig. 8:**
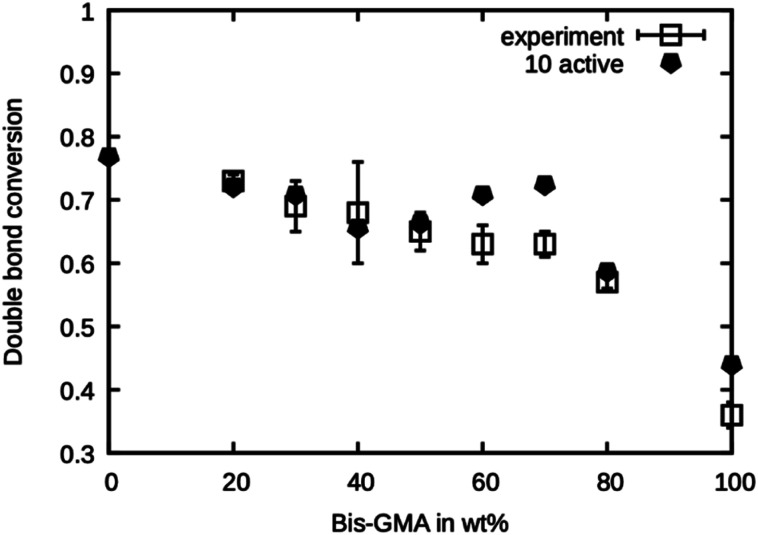
Double bond conversion as measured by Dewaele *et al.*^25^ compared to the double bond conversion of the model with ten active sites after the termination criterion was reached over the Bis-GMA content.

This series of simulations were repeated using only ten active sites. Fewer active sites yield a lower probability of finding a reaction partner, especially when the mobility is limited by the onset of gelation in a later stage of the curing process. The results are presented in Fig. 8. It can be seen that the double bond conversion is generally lower than in the case of 20 active sites and is in excellent agreement with the experimental values. Only for 60 wt% and 70 wt% Bis-GMA content, the values are too high.

Note that the presented method does not account for any termination reactions that might lower the degree of polymerization. This model only takes propagation into account. Termination only happens when the mobility is reduced so much that no reaction occurs in the specified amount of time. From the excellent agreement of the simulation with ten active sites, it can be concluded that this is the number of ‘effective’ active sites in the system. To relate the ‘effective’ number of sites to the concentration of initiator in the real system, a precise knowledge of the kinetics of the termination reactions would be necessary.

**Fig. 9:**
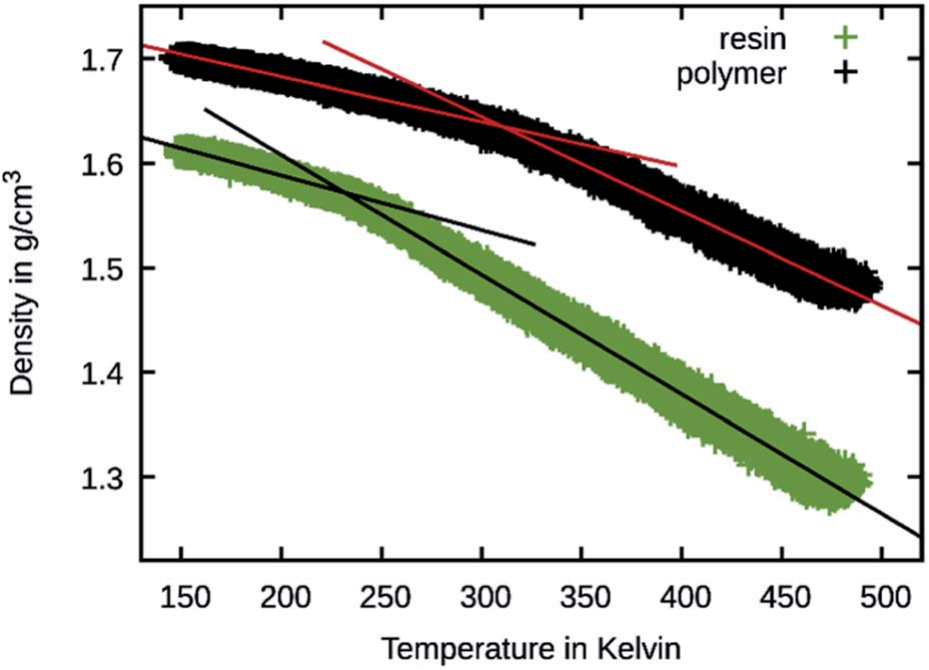
Temperature sweep results of 50 wt% Bis-GMA resin and polymer. The density is shown over the temperature together with linear fit functions.

To better judge the mobility change induced by the crosslinking, temperature sweeps were performed on the 50 wt% Bis-GMA resin and its crosslinked version to observe changes in the density slopes. This is a common method to estimate the glass transition temperature^27^ and was also previously performed using the Martini force field.^28^ The results are shown in Fig. 9. For estimating the glass transition temperature, the data was piecewise fitted using linear functions. The temperature at the intersection is 310 K for the polymer and 232 K for the resin. This indicates that during the polymerization simulation the resin transitions to a glassy polymer as the simulation temperature is below the estimated glass transition for the polymerized material.

## Conclusions

The goal of this work was to explore the use of the Martini coarse grain model for simulating photopolymerizable resins, which are used in multiple different industries. One major field of application is restorative dentistry. Thus, Bis-GMA – TEGDMA mixtures were chosen as a logical starting point. Both molecules were parametrized as outlined on the Martini web page.^17^ The bonded interactions were parametrized to fit the distributions of the all-atom simulations best. In Bis-GMA, the asymmetric dihedral angle configuration observed in the all-atom simulation was also reproduced in the coarse grain model. This was achieved while keeping the symmetry of all bonded interaction parameters.

Atom to bead mapping was done as recommended by the Martini authors.^14,23,24^ The obtained model reproduces resin densities reported in literature well. The use of standard Martini building blocks makes it easy to add additional molecules of interest later on and expand the set of possible resin formulations in the future.

Critical for the use of a photopolymer is the polymerization shrinkage and double bond conversion of the polymerized material. This is the case in restorative dentistry as well as in additive manufacturing. Our simulations show that the presented model can successfully predict polymerization shrinkage of various molecular mixtures. This information is valuable for the development of dental composites to assess the risk of debonding of filling and tooth^4,5^ or for 3D printing materials to avoid warpage or shrinkage stresses in additively manufactured parts.^7^ The use of a simulated polymerization also allows for an estimation of double bond conversions for different resin mixtures and different initiator concentrations. The presented method well reproduces double bond conversions for Bis-GMA – TEGDMA mixtures reported by Dewaele *et al.*^25^

## Author contributions

Alexander Hochwallner: conceptualization; methodology; software; formal analysis; investigation; writing – original draft; writing – review and editing; visualization. Jürgen Stampfl: conceptualization; writing – review and editing; supervision.

## Conflicts of interest

There are no conflicts to declare.

## Supplementary Material
